# Supplementary material for: Citrullination modulates antigen processing and presentation by revealing cryptic epitopes in rheumatoid arthritis
Source: Nat Commun. 2023 Feb 24;14:1061. doi: 10.1038/s41467-023-36620-y (PMC9958131; doi:10.1038/s41467-023-36620-y)
Supplement: Supplementary file 1 — Supplementary Information [file 41467_2023_36620_MOESM1_ESM.pdf]

SUPPLEMENTARY INFORMATION

Citrullination modulates antigen processing and presentation by revealing cryptic epitopes in rheumatoid arthritis

Ashley M. Curran[1], Alexander A. Girgis[1,2], Yura Jang[3†], Jonathan D. Crawford[1], Mekha A. Thomas[1], Ryan Kawalerski[2,4], Jeff Coller[4,5], Clifton O. Bingham III[1], Chan Hyun Na[3], Erika Darrah[1*]

Affiliations:
[1]Rheumatology, Johns Hopkins University School of Medicine, Baltimore, MD, USA
[2]Biomedical Engineering, Johns Hopkins University, Baltimore, MD, USA
[3]Neurology, Institute for Cell Engineering, Johns Hopkins University School of Medicine, Baltimore, MD, USA
[4]Molecular Biology and Genetics, Johns Hopkins University School of Medicine, Baltimore, MD, USA
[5]Biology, Johns Hopkins University, Baltimore, MD, USA

[†]Current affiliation: Laboratory of Immunology, Office of Biotechnology Products, Center for Drugs Evaluation and Research, Food and Drug Administration, Silver Spring, MD, USA

**Corresponding author—email: edarrah1@jhmi.edu*

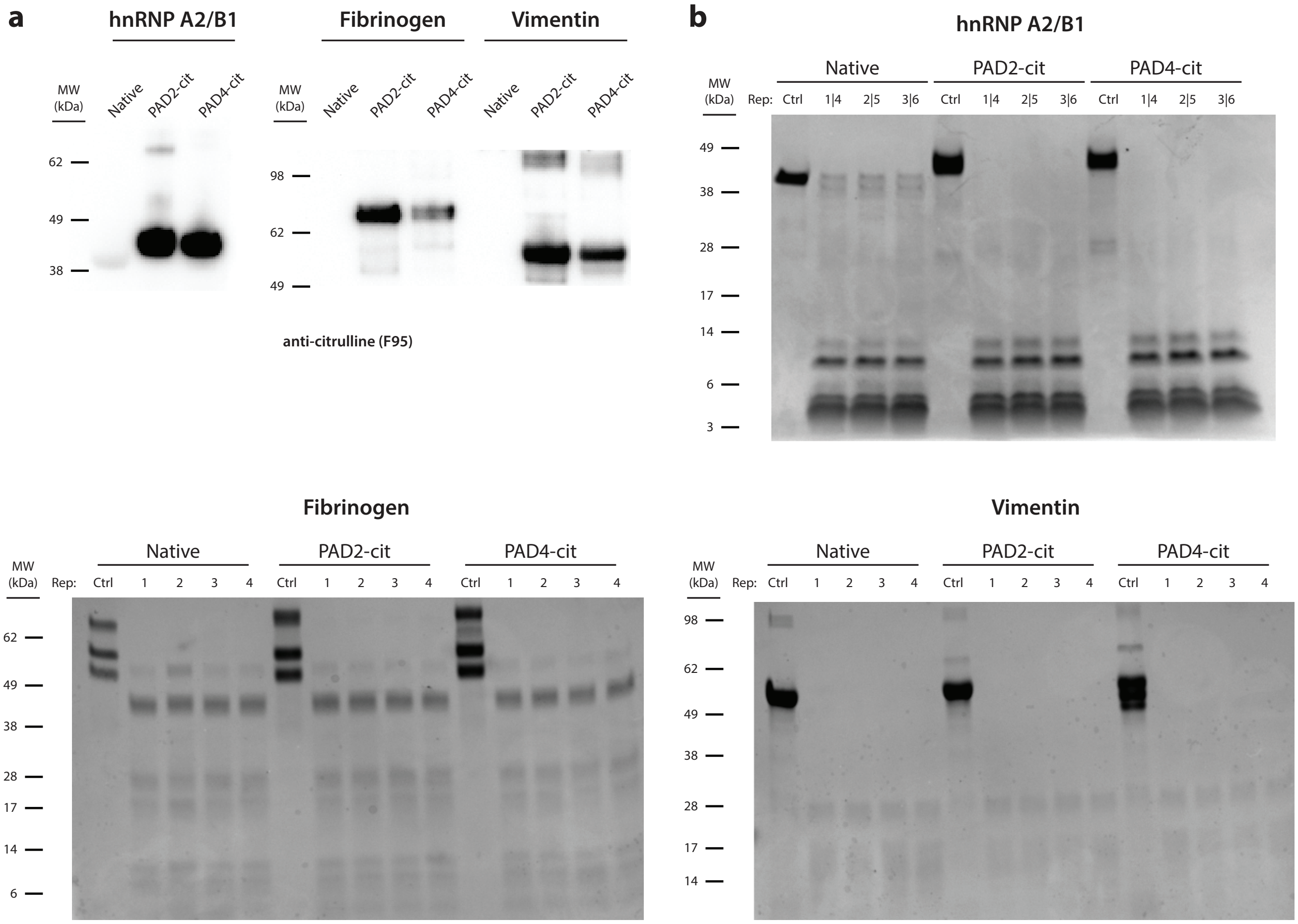

Supplementary Fig. 1 | Confirmation of autoantigen citrullination and digestion for ProtMap. a, Anti-citrulline (F95) Western blots to confirm citrullination of PAD2- and PAD4-citrullinated antigens prior to splitting samples for ProtMap. **b**, Coomassie Blue–stained gels of each antigen following ProtMap to confirm digestion. Replicates of hnRNP A2/B1 digestion were divided into two and analyzed as six separate replicates by mass spectrometry according to the labels. Estimated molecular weight markers shown for fibrinogen and vimentin.

PAD2-specific cit site   Cit site - both PADs   PAD4-specific cit site

a

```
> fibrinogen alpha chain

MFSMRIVCLVLSVVGTAWTADSGEGDFLAEGGGVRGPRVVERHQSACKDSDWPFCSDEDWNYKCPSGCRMKGLIDEVNQDFTNRINKLKNSLFEYQN
NKDSHSLTTNIMEILRGDFSSANNRDNTYNRVSEDLRSRIEVLKRKVIEKVQHIQLLQKNVRAQLVDMKRLEVDIDIKIRSCRGSCSRALAREVDLK
DYEDQQKQLEQVIAKDLLPSRDRQHLPLIKMKPVPDLVPGNFKSQLQKVPPEWKALTDMPQMRMELERPGGNEITRGGSTSYGTGSETESPRNPSSA
GSWNSGSSGPGSTGNRNPGSSGTGGTATWKPGSSGPGSTGSWNSGSSGTGSTGNQNPGSPRPGSTGTWNPGSSERGSAGHWTSESSVSGSTGQWHSE
SGSFRPDSPGSGNARPNNPDWGTFEEVSGNVSPGTRREYHTEKLVTSKGDKELRTGKEKVTSGSTTTTRRSCSKTVTKTVIGPDGHKEVTKEVVTSE
DGSDCPEAMDLGTLSGIGTLDGFRHRHPDEAAFFDTASTGKTFPGFFSPMLGEFVSETESRGSESGIFTNTKESSSHHPGIAEFPSRGKSSSYSKQF
TSSTSYNRGDSTFESKSYKMADEAGSEADHEGTHSTKRGHAKSRPVRDCDDVLQTHPSGTQSGIFNIKLPGSSKIFSVYCDQETSLGGWLLIQQRMD
GSLNFNRTWQDYKRGFGSLNDEGEGEFWLGNDYLHLLTQRGSVLRVELEDWAGNEAYAEYHFRVGSEAEGYALQVSSYEGTAGDALIEGSVEEGAEY
TSHNNMQFSTFDRDADQWEENCAEVYGGGWWYNNCQAANLNGIYYPGGSYDPRNNSPYEIENGVVWVSFRGADYSLRAVRMKIRPLVTQ

> fibrinogen beta chain

MKRMVSWSFHKLKTMKHLLLLLLCVFLVKSQGVNDNEEGFFSARGHRPLDKKREEAPSLRPAPPPISGGGYRARPAKAAATQKKVERKAPDAGGCLH
ADPDLGVLCPTGCQLQEALLQQERPIRNSVDELNNNVEAVSQTSSSSFQYMYLLKDLWQKRQKQVKDNENVVNEYSSELEKHQLYIDETVNSNIPTN
LRVLRSILENLRSKIQKLESDVSAQMEYCRTPCTVSCNIPVVSGKECEEIIRKGGETSEMYLIQPDSSVKPYRVYCDMNTENGGWTVIQNRQDGSVD
FGRKWDPYKQGFGNVATNTDGKNYCGLPGEYWLGNDKISQLTRMGPTELLIEMEDWKGDKVKAHYGGFTVQNEANKYQISVNKYRGTAGNALMDGAS
QLMGENRTMTIHNGMFFSTYDRDNDGWLTSDPRKQCSKEDGGGWWYNRCHAANPNGRYYWGGQYTWDMAKHGTDDGVVWMNWKGSWYSMRKMSMKIR
PFFPQQ

> fibrinogen gamma chain

MSWSLHPRNLILYFYALLFLSSTCVAYVATRDNCCILDERFGSYCPTTCGIADFLSTYQTKVDKDLQSLEDILHQVENKTSEVKQLIKAIQLTYNPD
ESSKPNMIDAATLKSRKMLEEIMKYEASILTHDSSIRYLQEIYNSNNQKIVNLKEKVAQLEAQCQEPCKDTVQIHDITGKDCQDIANKGAKQSGLYF
IKPLKANQQFLVYCEIDGSGNGWTVFQKRLDGSVDFKKNWIQYKEGFGHLSPTGTTEFWLGNEKIHLISTQSAIPYALRVELEDWNGRTSTADYAMF
KVGPEADKYRLTYAYFAGGDAGDAFDGFDFGDDPSDKFFTSHNGMQFSTWDNDNDKFEGNCAEQDGSGWWMNKCHAGHLNGVYYQGGTYSKASTPNG
YDNGIIWATWKTRWYSMKKTTMKIIPFNRLTIGEGQQHHLGGAKQVRPEHPAETEYDSLYPEDDL
```

b

```
> hnRNP A2/B1

MEKTLETVPLERKKREKEQFRKLFIGGLSFETTEESLRNYYEQWGKLTDCVVMRDPASKRSRGFGFVTFSSMAEVDAAMAARPHSIDGRVVEPKRAV
AREESGKPGAHVTVKKLFVGGIKEDTEEHHLRDYFEEYGKIDTIEIITDRQSGKKRGFGFVTFDDHDPVDKIVLQKYHTINGHNAEVRKALSRQEMQ
EVQSSRSGRGGNFGFGDSRGGGGNFGPGPGSNFRGGSDGYGSGRGFGDGYNGYGGGPGGGNFGGSPGYGGGRGGYGGGGPGYGNQGGGYGGGYDNYG
GGNYGSGNYNDFGNYNQQPSNYGPMKSGNFGGSRNMGGPYGGGNYGPGGSGGSGGYGGRSRY
```

c

```
> vimentin

MSTRSVSSSSYRRMFGGPGTASRPSSSRSYVTTSTRTYSLGSALRPSTSRSLYASSPGGVYATRSSAVRLRSSVPGVRLLQDSVDFSLADAINTEFK
NTRTNEKVELQELNDRFANYIDKVRFLEQQNKILLAELEQLKGQGKSRLGDLYEEEMRELRRQVDQLTNDKARVEVERDNLAEDIMRLREKLQEEML
QREEAENTLQSFRQDVDNASLARLDLERKVESLQEEIAFLKKLHEEEIQELQAQIQEQHVQIDVDVSKPDLTAALRDVRQQYESVAAKNLQEAEEWY
KSKFADLSEAANRNNDALRQAKQESTEYRRQVQSLTCEVDALKGTNESLERQMREMEENFAVEAANYQDTIGRLQDEIQNMKEEMARHLREYQDLLN
VKMALDIEIATYRKLLEGEESRISLPLPNFSSLNLRETNLDSLPLVDTHSKRTLLIKTVETRDGQVINETSQHHDDLE
```

d

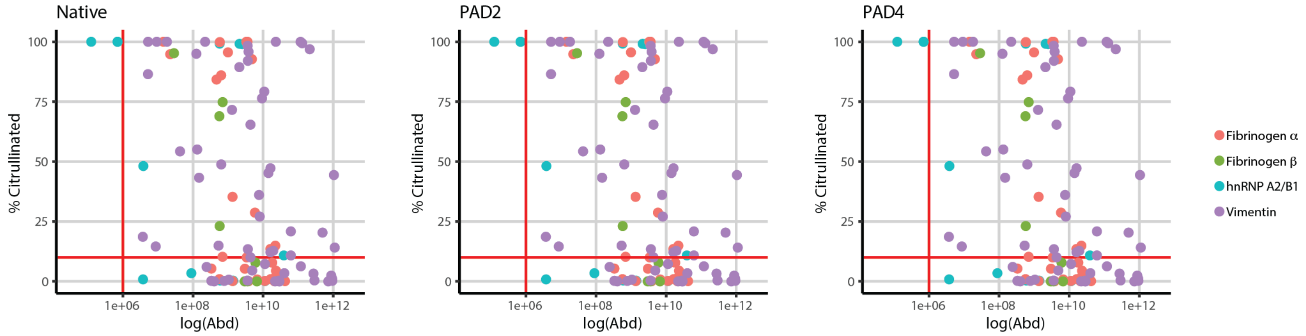

Supplementary Fig. 2 | Citrullination site mapping by mass spectrometry. a-c, Citrullination sites in PAD2- and PAD4-citrullinated antigens identified by mass spectrometry, color-coded based on the PAD enzymes that generated them according to the legend. **d**, The abundance of all arginine residues with detected citrullination in MS data plotted against the frequency that residue was citrullinated. Red lines indicate final inclusion thresholds.

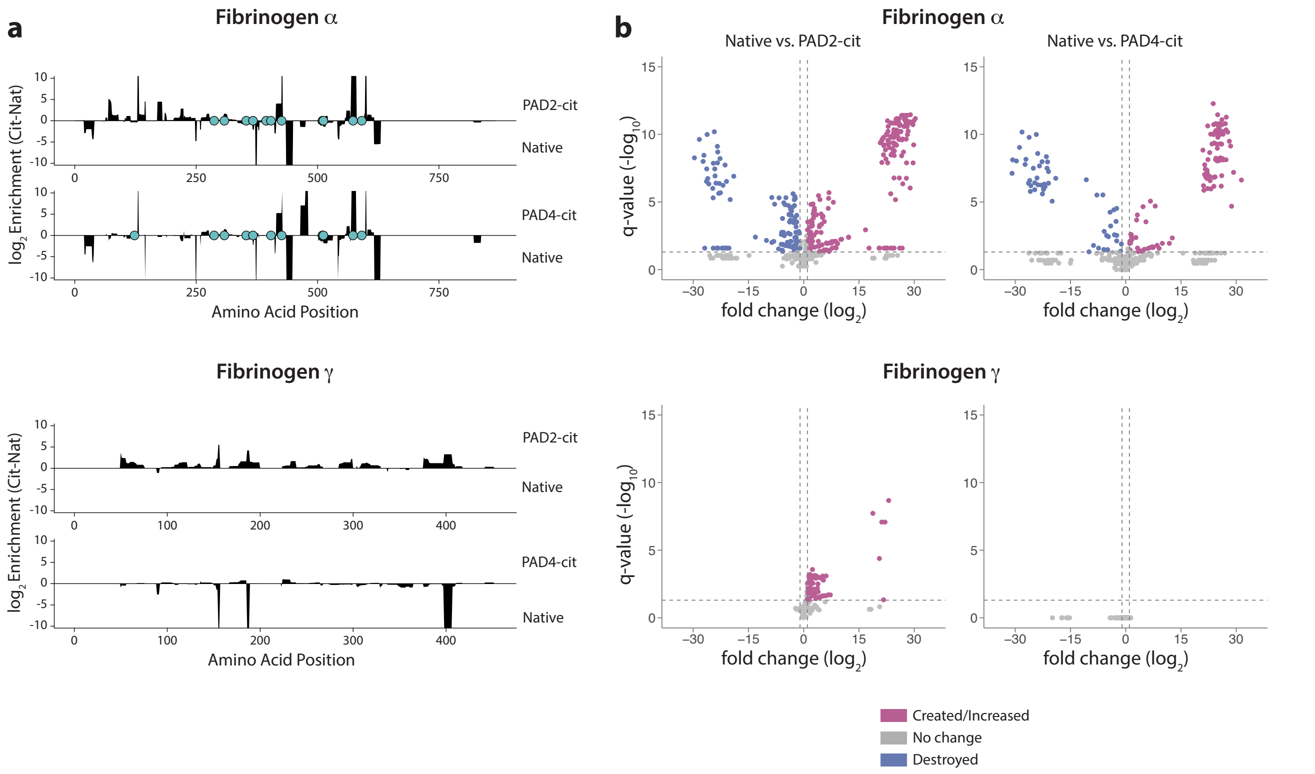

Supplementary Fig. 3 | Citrullination alters antigen processing of the fibrinogen α and γ chains, resulting in the simultaneous generation of cryptic peptides and destruction of previously dominant peptides. a, $\log_2$ enrichment of peptides from proteolytic mapping with cathepsins BSH across the primary amino acid sequence of the fibrinogen α and γ chains. Amino acid positions with positive values are enriched in the PAD2- or PAD4-citrullinated (cit) samples, while those with negative values are reduced by citrullination. Citrulline residues are denoted by filled blue circles on x-axes. **b**, Volcano plots representing peptides with significantly altered abundance. Vertical lines denote a fold change of 2, and the horizontal line denotes a cutoff of 0.05 for the FDR-corrected *P* value (*q* value), calculated by paired two-sided Student's t-tests. Peptides to the left or right of vertical dashed lines and above horizontal dashed line are deemed to be significantly different between the groups. Proteolytic mapping was performed in replicates of 4 (fibrinogen and vimentin) or 6 (hnRNP A2/B1).

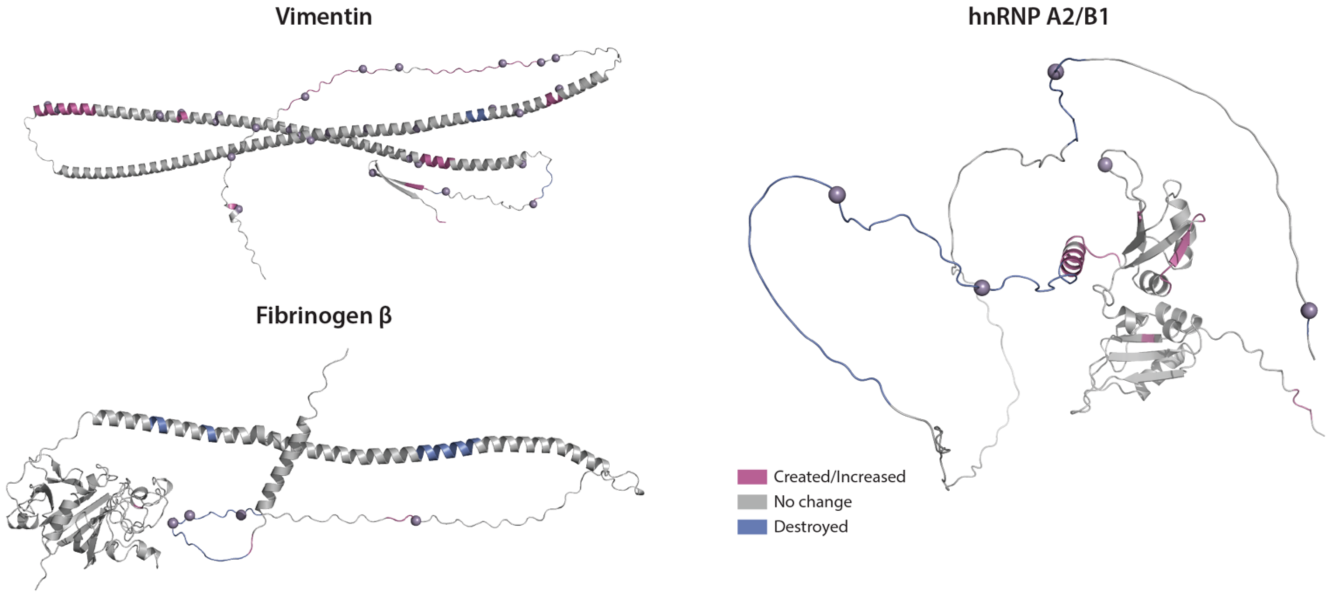

Supplementary Fig. 4 | Citrullination impacts protein dynamics of the fibrinogen α and γ chains by promoting the destabilization of protein folding. Predicted PAD4-citrullinated structures with significantly changed regions color-coded according to legend and PAD4 citrullination sites denoted as purple spheres.

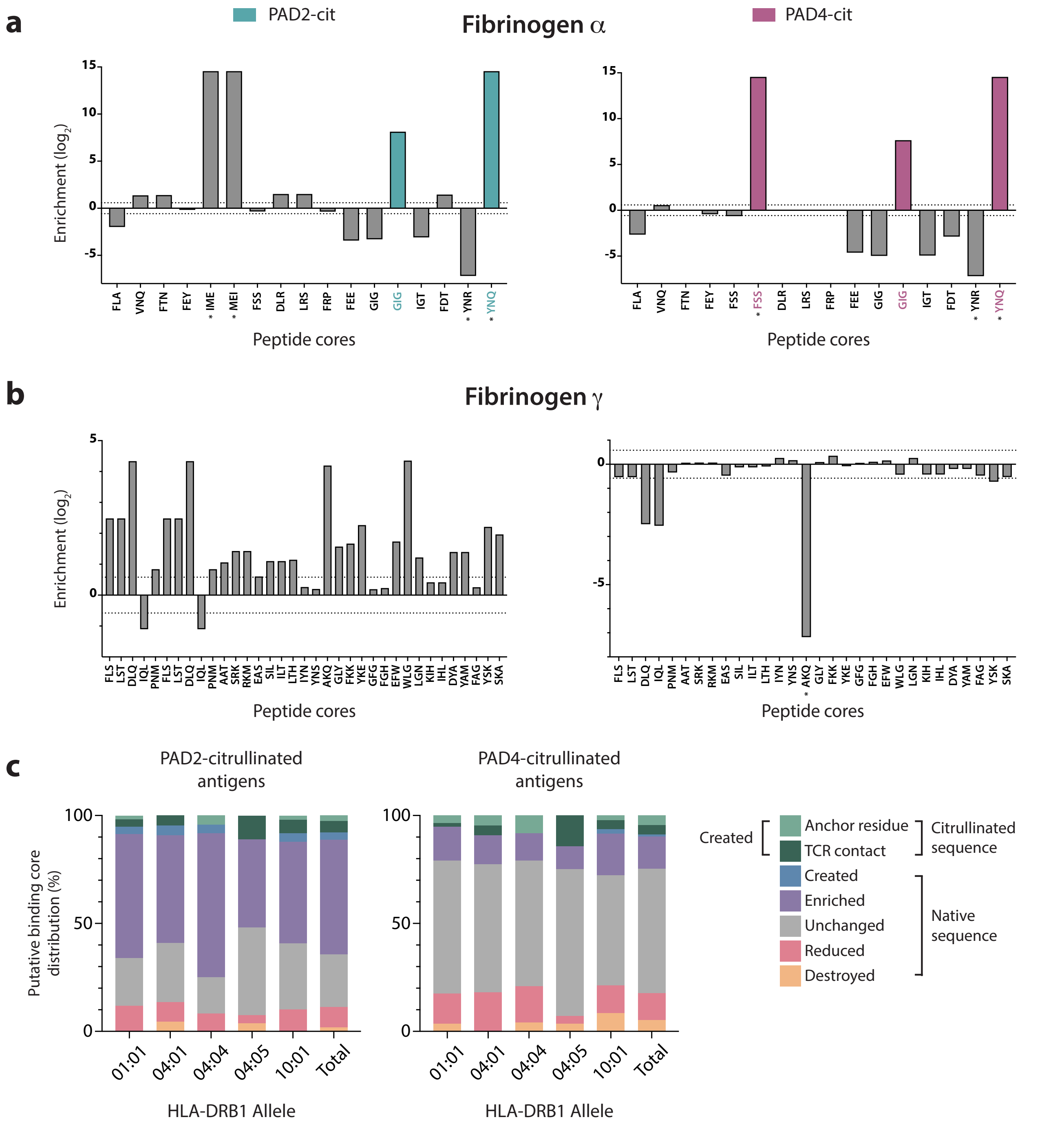

Supplementary Fig. 5 | Peptide cores from the fibrinogen α and γ chains predicted to bind with high affinity to RA-associated HLA-DR molecules. a-b, $\log_2$ enrichment of high-affinity binding cores (<500 nM) the fibrinogen α and γ chains predicted by the NetMHCII-2.3 algorithm, denoted by the first three amino acids of the core. Cores with positive values are enriched in the PAD2- or PAD4-citrullinated (cit) samples, while those with negative values are reduced by citrullination. * denotes a uniquely created or destroyed peptide, and color-coded cores are those that contained a citrullination site. **c**, Proportion of all putative binding cores belonging to several categories (given by legend) denoting the behavior of each core in response to citrullination (from Fig. 4), separated by shared epitope allele.

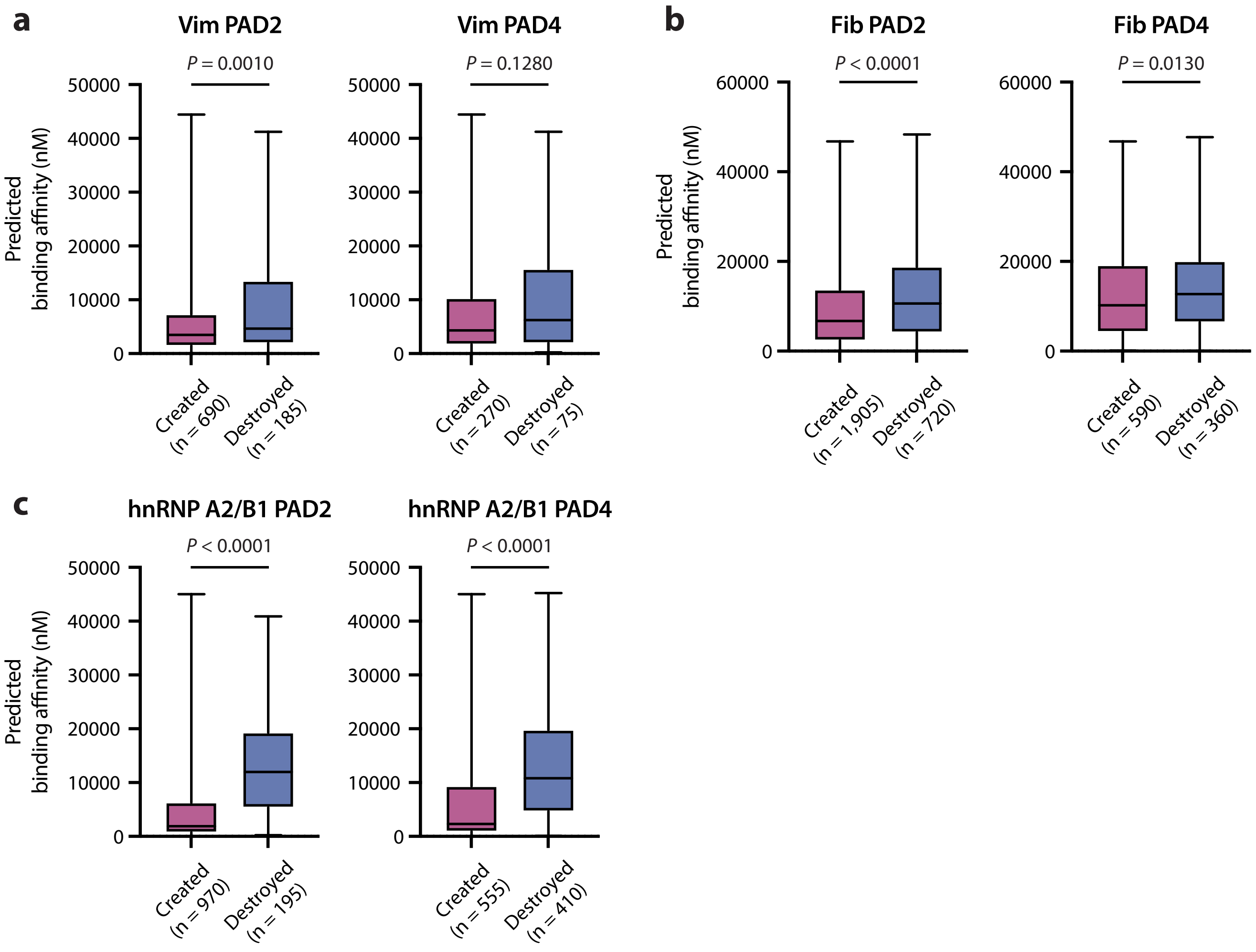

Supplementary Fig. 6 | Median binding affinity of created and destroyed peptide repertoires from ProtMap to SE HLA-DR variants. a-c, Median binding affinities of created and destroyed peptide repertoires derived from proteolytic mapping of the three model RA autoantigens – vimentin (vim), fibrinogen (fib), and hnRNP A2/B2 - to SE HLA-DR molecules (*01:01, *04:01, *04:04, *04:05, and *10:01) as predicted by NetMHCII-2.3 binding prediction algorithm. Predicted binding affinities of the created versus destroyed repertoires for each antigen were compared using non-parametric, two-tailed Mann Whitney *U* tests. The boxes represent the 25th to 75th percentiles, the center lines represent the median, and the whiskers denote the 5th to 95th percentiles. *P* values ≤ 0.05 were considered significant.

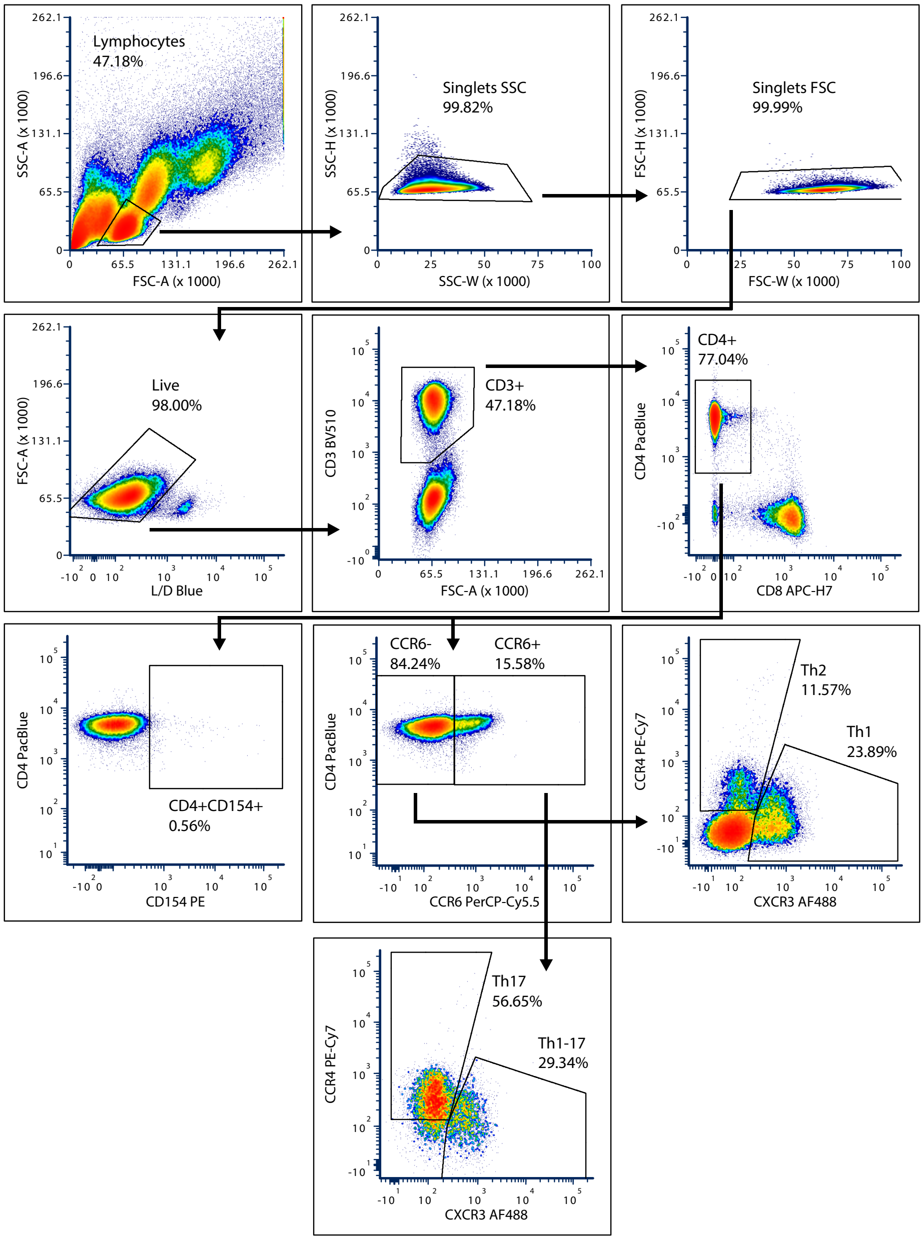

Supplementary Fig. 7 | CD4⁺ T cell stimulation assay flow cytometry gating strategy. Flow cytometry gating strategy for CD4⁺ T cell CD154 and helper subset analyses from Fig. 6.

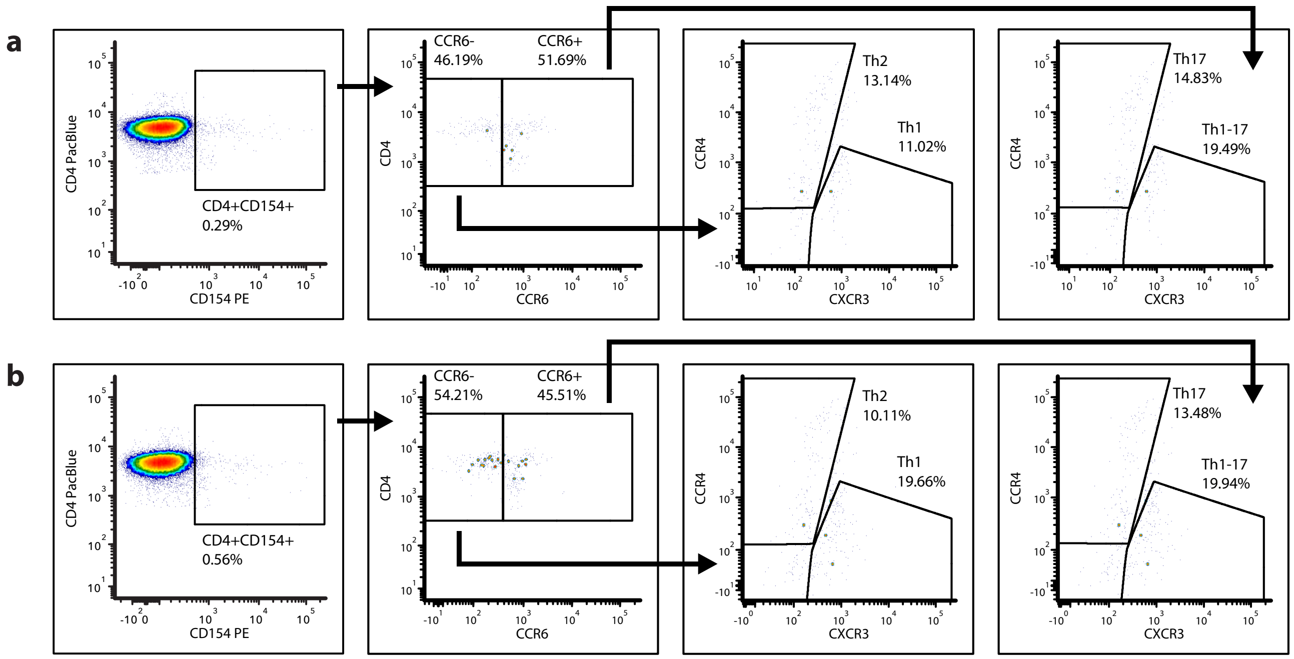

Supplementary Fig. 8 | | CD4$^+$ T cell stimulation assay representative flow cytometry plots. Representative flow cytometry plots for CD4$^+$ T cell CD154 and helper subset analyses from Fig. 6. **a,** Representative plots from an ACPA$^+$ RA patient stimulated with media alone to measure background activation. **b,** Representative plots from an ACPA$^+$ RA patient stimulated with a created fibrinogen peptide.

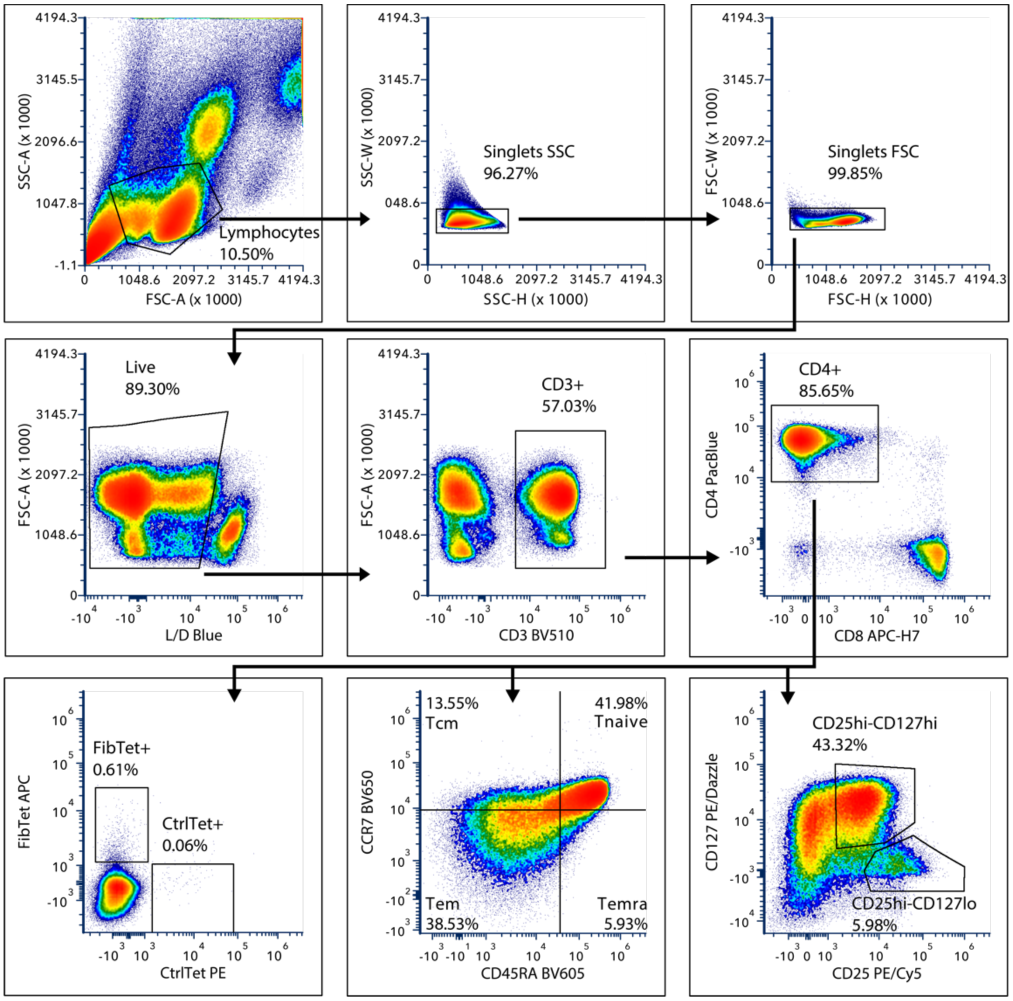

Supplementary Fig. 9 | CD4$^+$ T cell MHC class II tetramer assay flow cytometry gating strategy. Flow cytometry gating strategy for CD4$^+$ T cell tetramer and effector phenotype analyses from Fig. 7.

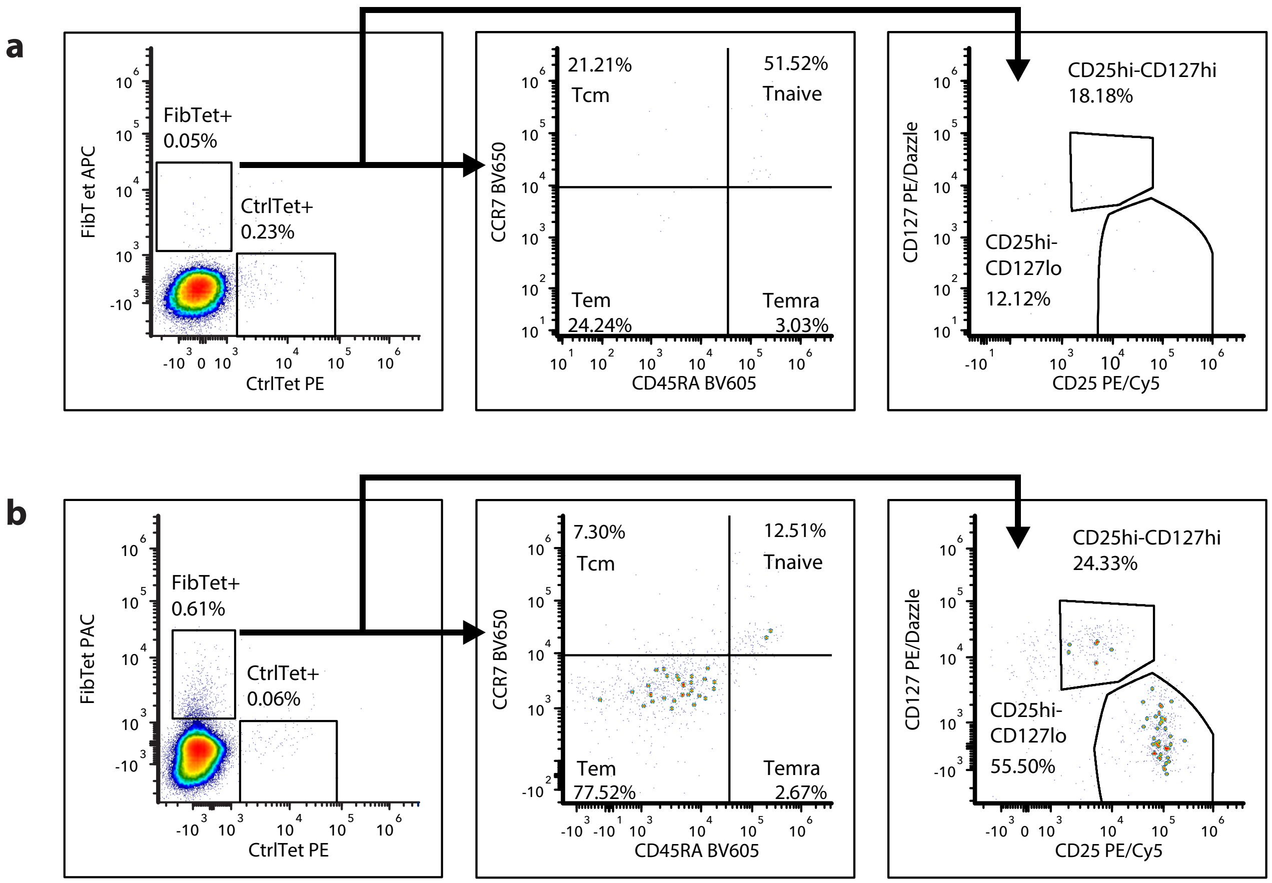

Supplementary Fig. 10 | CD4$^+$ T cell MHC class II tetramer assay representative flow cytometry plots. Representative flow cytometry plots for CD4$^+$ T cell tetramer and effector phenotype analyses from Fig. 7. **a,** Representative plots from a FibTet-negative ACPA$^-$ RA patient. **b,** Representative plots from a FibTet-positive ACPA$^+$ RA patient.

Supplementary Table 1 | ProtMap changed residues and regions

Antigen	PAD condition	# citrulline residues	citrullination (%)	# changed residues	changed residues (%)	# changed peptides		changed peptides (%)	
						Cr	De	Cr	De
Fibrinogen α	PAD2	12	1.39	211.00	24.36	203	116	35.80	20.46
	PAD4	12	1.39	192.00	22.17	101	54	18.10	9.68
Fibrinogen β	PAD2	5	1.02	153.00	31.16	90	31	29.03	10.00
	PAD4	4	0.81	46.00	9.37	23	22	7.44	7.12
Fibrinogen γ	PAD2	0	0.00	122.00	26.27	89	0	33.58	0.00
	PAD4	0	0.00	22.00	4.86	0	0	0.00	0.00
Vimentin	PAD2	26	5.58	170.00	36.48	139	38	42.38	11.59
	PAD4	30	6.44	87.00	18.67	55	15	17.08	4.66
hnRNP A2/B1	PAD2	8	2.27	110.00	31.16	194	40	48.62	10.03
	PAD4	5	1.42	88.00	24.93	111	82	24.61	18.18

Cr = created, De = destroyed

Supplementary Table 2 | Structural analysis scores

Antigen	% arginine	PAD2 change score	PAD4 change score	RMSD PAD2	RMSD PAD4	RMSD PAD2/4	TM-score PAD2	TM-score PAD4	TM-score PAD2/4
Fibrinogen α	6.3	56.26	27.78	-	-	-	-	-	-
Fibrinogen β	5.8	39.03	14.56	5.90	5.94	7.01	0.89	0.88	0.88
Fibrinogen γ	2.6	33.58	0.00	-	-	-	-	-	-
Vimentin	9.2	53.96	21.74	12.16	13.96	15.36	0.75	0.75	0.65
hnRNP A2/B1	7.1	58.65	42.79	17.12	26.96	26.46	0.67	0.60	0.62

Supplementary Table 3 | Putative binding core classifications

Core Category		PAD2		PAD4	
		# unique cores	% total cores	# unique cores	% total cores
Citrulline	Anchor residue	3	2.61	5	4.42
	TCR contact	6	5.22	5	4.42
Native	Created	4	3.48	1	0.88
	Enriched	61	53.04	17	15.04
	Unchanged	28	24.35	65	57.52
	Reduced	11	9.57	14	12.39
	Destroyed	2	1.74	6	5.31

Supplementary Table 4 | NAPA peptide sequences

Chain	Residues	NATIVE FIBRINOGEN	PAD2-CITRULLINATED	PAD4-CITRULLINATED
Alpha Chain	D21-R35	DSGEGDFLAEGGVR	[DSGEGDFLAEGGVR]	[DSGEGDFLAEGGVR]
	L155-E170	[KNVRAQLVDMKR]	KNVRAQLVDMKR	LQKNVRAQLVDMKRLE
	N361-H387	NPGSSERGSAGHWTSESSVSGSTG[QWH]	[NPGSSER]GSAGHWTSESSVSGSTG	[NPGSSER]GSAGHWTSESSVSGSTG[QWH]
		GSAGHWTSESSVSGSTG	SAGHWTSESSVSGSTGQWH	SAGHWTSESSVSGSTG
		SAGHWTSESSVSGSTG	SAGHWTSESSVSGST	AGHWTSESSVSGSTG
		AGHWTSESSVSG	AGHWTSESSVSGSTG	AGHWTSESSVSGST
		AGHWTSESSVSGST	AGHWTSESSVSGST	GHWTSESSVSGSTG
		GHWTSESSVSGSTG	AGHWTSESSVSG	
			GHWTSESSVSGSTG	
			GHWTSESSVSGST	
	L537-S551	LGEFVSETESRGSES	[LGEFVSETESRGSES]	[LGEFVSETESRGSES]
		LGEFVSETESRGSE		
		LGEFVSETESRG		
		LGEFVSETESR		
	Y579-S594	YSKQFTSSTSYNRGDS	[YSKQFTSSTSYNRGDS]	[YSKQFTSSTSYNRGDS]
Beta Chain	L176-T193	[LEKHQLYIDETVNSNIPT]	EKHQLYIDETVNSNIPT	LEKHQLYIDETVNSNIPT
				EKHQLYIDETVNSNIPT
	K247-Y266	[RKGGE]TSEMYLIQPDSSVKPY	[R]KGGETSEMYLIQPDSSVKPY	[R]KGGETSEMYLIQPDSSVKPY
			GGETSEMYLIQPDSSVKP	GETSEMYLIQPDSSVKPY
			GGETSEMYLIQPDSSVKPY	GGETSEMYLIQPDSSVKP
			GETSEMYLIQPDSSVKPY	GETSEMYLIQPDSSVKPY
			TSEMYLIQPDSSVKPY	TSEMYLIQPDSSVKPY
			SEMYLIQPDSSVKPY	SEMYLIQPDSSVKPY
	N363-G377	[NEANKYQISVNKYRG]	NEANKYQISVNKYRG	EANKYQISVNKYRG
Gamma Chain	Y94-M115	[Y]NPDESSKPNMIDAATLKSRKM	YNPDESSKPNMIDAATLKSRK	YNPDESSKPNMIDAATLKSRK
		NPDESSKPNMIDAATLKSRK	NPDESSKPNMIDAATLKSRKM	NPDESSKPNMIDAATLKSRKM
		SKPNMIDAATLKSRKM	NPDESSKPNMIDAATLKSRK	NPDESSKPNMIDAATLKSRK
		SKPNMIDAATLKSRK	SKPNMIDAATLKSRK	SKPNMIDAATLKSRKM
		KPNMIDAATLKSRK	KPNMIDAATLKSRK	SKPNMIDAATLKSRK
				SKPNMIDAATLKSR
				KPNMIDAATLKSRK
	Y375-W395	YQGGTYSKASTPNGYDNG[IIW]	YQGGTYSKASTPNGYDNGIIW	YQGGTYSKASTPNGYDNGIIW
		GGTYSKASTPNGYD	YQGGTYSKASTPNGYDNGI	YQGGTYSKASTPNGYDNG
			YQGGTYSKASTPNGYDNG	YQGGTYSKASTPNGYDN
			YQGGTYSKASTPNGYDN	QGGTYSKASTPNGYDNGI
			QGGTYSKASTPNGYDNG	QGGTYSKASTPNGYDNG
			QGGTYSKASTPNGYDN	QGGTYSKASTPNGYDN
			GGTYSKASTPNGYDNGI	GGTYSKASTPNGYDNGIIW
			GGTYSKASTPNGYDNG	GGTYSKASTPNGYDNGI
			GGTYSKASTPNGYDN	GGTYSKASTPNGYDNG
			GGTYSKASTPNGYD	GGTYSKASTPNGYDN
			GTYSKASTPNGYDNG	GGTYSKASTPNGYD
			GTYSKASTPNGYDN	

Grey amino acids or peptides do not appear in a given peptide or sample, respectively.

Teal peptides were uniquely created in the citrullinated sample.

Red arginines were citrullinated in in vitro proteolytic mapping data from the same antigens.

Supplementary Table 5 | ProImmune REVEAL® Peptide Binding Scores

Peptide Category	Peptide #	Peptide ID	Sequence	Chain	REVEAL® Score
Created	1	Fib_A2	LQKNVRAQLVDMKRLE	Alpha	12.1
	2	Fib_B1	LEKHQLYIDETVNSNIPT	Beta	55.8
	3	Fib_B4	NEANKYQISVNKYRG	Beta	69.5
	4	Fib_G2	YQGGTYSKASTPNGYDNGIIW	Gamma	26.2
No Change	5	Fib_A4	GSAGHWTSESSVSGSTG	Alpha	57.2
	6	Fib_B3	TSEMYLIQPDSSVKPY	Beta	8.2
	7	Fib_G1	YNPDESSKPNMIDAATLKSRKM	Gamma	93.3
Destroyed	8	Fib_A1	DSGEGDFLAEGGVR	Alpha	19.2
	9	Fib_A5	LGEFVSETESRGSES	Alpha	1.1
	10	Fib_A6	YSKQFTSSTSYNRGDS	Alpha	108.7

Supplementary Table 6 | RA Patient Demographic Characteristics

Variable	T cell stimulations		Tetramer assays	
	ACPA$^+$	ACPA$^-$	ACPA$^+$	ACPA$^-$
Total number	10	8	18	10
Female, no. (%)	6 (60)	8 (100)	15 (83)	10 (100)
Age (years), mean ± SD	63.43 ± 12.28	67.87 ± 17.25	61.67 ± 11.60	67.45 ± 15.75
Disease duration (years), mean ± SD	21.80 ± 16.82	14.63 ± 5.37	15.28 ± 7.51	15.00 ± 4.71

Supplementary Table 7 | T cell stimulation peptide sequences

Peptide Category	Peptide #	Peptide ID	Sequence	Chain	Residues
Created	1	Fib_A2	LQKNVRAQLVDMKRLE	Alpha	L155-E170
	2	Fib_B1	LEKHQLYIDETVNSNIPT	Beta	L176-T193
	3	Fib_B4	NEANKYQISVNKYRG	Beta	N363-G377
	4	Fib_G2	YQGGTYSKASTPNGYDNGIIW	Gamma	Y375-W395
No Change	5	Fib_A4	GSAGHWTSESSVSGSTG	Alpha	G368-G384
	6	Fib_B3	TSEMYLIQPDSSVKPY	Beta	T251-Y266
	7	Fib_G1	YNPDESSKPNMIDAATLKSRKM	Gamma	Y94-M115
Destroyed	8	Fib_A1	DSGEGDFLAEGGVR	Alpha	D21-R35
	9	Fib_A5	LGEFVSETESRGSES	Alpha	L537-S551
	10	Fib_A6	YSKQFTSSTSYNRGDS	Alpha	Y579-S594

Supplementary Table 8 | MHC Class II Tetramers

Tetramer #	Peptide ID	Sequence	Fluorophore
1	Fib_A2	QLLQKNVRAQLVDMKRLE	APC
2	Fib_B1	LEKHQLYIDETVNSNIPT	
3	Fib_B4	NEANKYQISVNKYRG	
4	Tet	TKIYSYFPSVISKV	PE
5	Flu	PKYVKQNTLKLAT	
6	CLIP	PVSKMRMATPLLMQA	

Tet = tetanus, Flu = influenza
