# Supplementary material for: Citrullination modulates antigen processing and presentation by revealing cryptic epitopes in rheumatoid arthritis
Source: Nat Commun. 2023 Feb 24;14:1061. doi: 10.1038/s41467-023-36620-y (PMC9958131; doi:10.1038/s41467-023-36620-y)
Supplement: Supplementary file 3 — Reporting Summary [file 41467_2023_36620_MOESM3_ESM.pdf]

nature portfolio

Corresponding author(s): Erika Darrah
Last updated by author(s): Jan 24, 2023

Reporting Summary

Nature Portfolio wishes to improve the reproducibility of the work that we publish. This form provides structure for consistency and transparency in reporting. For further information on Nature Portfolio policies, see our Editorial Policies and the Editorial Policy Checklist.

Statistics

For all statistical analyses, confirm that the following items are present in the figure legend, table legend, main text, or Methods section.

n/a	Confirmed	
☐	☒	The exact sample size (*n*) for each experimental group/condition, given as a discrete number and unit of measurement
☐	☒	A statement on whether measurements were taken from distinct samples or whether the same sample was measured repeatedly
☐	☒	The statistical test(s) used AND whether they are one- or two-sided<br>*Only common tests should be described solely by name; describe more complex techniques in the Methods section.*
☒	☐	A description of all covariates tested
☐	☒	A description of any assumptions or corrections, such as tests of normality and adjustment for multiple comparisons
☐	☒	A full description of the statistical parameters including central tendency (e.g. means) or other basic estimates (e.g. regression coefficient) AND variation (e.g. standard deviation) or associated estimates of uncertainty (e.g. confidence intervals)
☐	☒	For null hypothesis testing, the test statistic (e.g. *F*, *t*, *r*) with confidence intervals, effect sizes, degrees of freedom and *P* value noted<br>*Give P values as exact values whenever suitable.*
☒	☐	For Bayesian analysis, information on the choice of priors and Markov chain Monte Carlo settings
☒	☐	For hierarchical and complex designs, identification of the appropriate level for tests and full reporting of outcomes
☒	☐	Estimates of effect sizes (e.g. Cohen's *d*, Pearson's *r*), indicating how they were calculated

Our web collection on statistics for biologists contains articles on many of the points above.

Software and code

Policy information about availability of computer code

Data collection	Proteome Discoverer (version 2.4.1.15, Thermo Scientific) and PEAKS (version 7, Bioinformatic Solutions) were used for the identification and quantification of proteins and peptides during mass spectrometry for proteolytic mapping and NAPA, respectively. The NetMHCII-2.3 peptide binding affinity prediction algorithm (DTU Health Tech; https://services.healthtech.dtu.dk/service.php?NetMHCII-2.3) was used to predict putative peptide binding cores to RA-associated SE HLA-DR molecules.<br><br>Predicted native and citrullinated protein structures for vimentin, fibrinogen (beta and gamma), and hnRNPA2/B1 were generated with AlphaFold v2.0 (installed as of commit '1d43aaf' from https://github.com/deepmind/alphafold). FASTAs for native and simulated citrullinated proteins were folded with the AlphaFold Docker script with the following parameters: "--max_template_date=2020-05-14" and "--db_preset=reduced_ dbs" in accordance with recommendations from the AlphaFold documentation.<br><br>FACSDiva version 6.0 (BD Biosciences) or SpectroFlo version 3.0.3 (Cytek Biosciences) software were used to collect flow cytometry data.
Data analysis	The code used in this study is available at https://github.com/DarrahLab/Curran-et-al-2022_Cit_AgProc (https://doi.org/10.5281/zenodo.7566507). Data analysis was performed in R, version 3.6.3.59, or Microsoft Excel, version 16.69.1 for Mac. All statistical analyses were performed using GraphPad Prism, version 9.3.0, or R, version 3.6.3.59. Protein structures were analyzed using PyMOL Molecular Graphics System, Version 2.4.2 (Schrödinger, LLC) and the Zhang Lab TM-align online algorithm (https://zhanggroup.org/TM-align/). Flow data was analyzed using FCS Express 7 version 7.12.0009 (De Novo Software). Cytokine assay analysis was performed using the MSD DISCOVERY WORKBENCH Desktop Analysis Software version 4.0 (Meso Scale Diagnostics; MSD).

For manuscripts utilizing custom algorithms or software that are central to the research but not yet described in published literature, software must be made available to editors and reviewers. We strongly encourage code deposition in a community repository (e.g. GitHub). See the Nature Portfolio guidelines for submitting code & software for further information.

Data

Policy information about availability of data

All manuscripts must include a data availability statement. This statement should provide the following information, where applicable:

- Accession codes, unique identifiers, or web links for publicly available datasets
- A description of any restrictions on data availability
- For clinical datasets or third party data, please ensure that the statement adheres to our policy

The UniProt database (https://www.uniprot.org/) was used for protein identification from the mass spectrometry data. All remaining data generated or analyzed during this study are provided in the Supplementary Data 1 and Source Data files.

Human research participants

Policy information about studies involving human research participants and Sex and Gender in Research.

Reporting on sex and gender	84% of the samples used in our study were collected from female RA patients, which is a reflection of RA demographics in our total patient cohort and in the general population. Given the large proportion of females, our study was not sufficiently powered to disaggregate our data by sex.
Population characteristics	Participants in this study were recruited from a longitudinal cohort of rheumatoid arthritis patients at the Johns Hopkins Arthritis Center. All patients participating in this study met the 2010 ACR-EULAR Classification Criteria for Rheumatoid Arthritis or were diagnosed with RA by a board-certified rheumatologist. Participants were classified based on SE and/or ACPA status for T cell stimulation assays. HLA-DRB1 genotyping was performed at the Johns Hopkins University Immunogenetics Laboratory by next-generation sequencing from flash-frozen cell pellets, and SE+ individuals were designated as those with at least one of the following SE alleles: HLA-DRB1*01:01, *04:01, *04:04, or *04:05. ACPA+ and ACPA– patients were selected based on a review of the clinical record and, when available, CCP3 values collected by the Johns Hopkins Rheumatic Disease Research Core Center (RDRCC). CCP3 values were measured in serum from patients for whom no CCP2 or CCP3 value was available from the clinical record or the RDRCC database (Quanta Lite CCP3 IgG ELISA, Inova Diagnostics). Patient population characteristics (i.e., age, sex, and disease duration) are provided in Supplementary Table 6.
Recruitment	Patients were recruited for this study from a larger observational registry study of RA ongoing in the Johns Hopkins Arthritis Center. For the parent study, every effort is made to include participants who reflect the demographic characteristics of the patient population in Baltimore with regard to sex and race. For this manuscript, patients were selected from this cohort based on anti-CCP status and HLA status. We did not restrict inclusion based on race, age, or sex, and there were no known self-selection or other biases present.
Ethics oversight	The study was approved by the Johns Hopkins Institutional Review Board, and all patients provided written informed consent. Patients did not receive compensation for their participation.

Note that full information on the approval of the study protocol must also be provided in the manuscript.

Field-specific reporting

Please select the one below that is the best fit for your research. If you are not sure, read the appropriate sections before making your selection.

[x] Life sciences [ ] Behavioural & social sciences [ ] Ecological, evolutionary & environmental sciences

For a reference copy of the document with all sections, see nature.com/documents/nr-reporting-summary-flat.pdf

Life sciences study design

All studies must disclose on these points even when the disclosure is negative.

Sample size	Proteolytic mapping experiments were done in replicates of at least four per antigen to ensure sufficient biological replicates for comparison and allow for a possible outlier sample to be excluded if necessary; however, no samples were excluded from analysis. NAPA was performed using cells from a SE+ donor with three forms of fibrinogen, and over 4000 unique peptides were recovered. ProImmune binding assays were performed on all 13 peptides identified by NAPA. No sample size calculation was required due to the nature of these experiments. T cell stimulation, tetramer, and MSD cytokine secretion assays were performed on 10 or more donors per condition when possible, which we predicted would provide >80% power to detect a significant difference between groups at an alpha of 0.05 based on previously published peptide-specific T cell detection assays using rheumatic patient cells in our lab (Tiniakou et al. 2020, DOI: 10.1002/art.41248).
Data exclusions	No data were excluded.
Replication	Proteolytic mapping experiments were performed in 4-6 biological replicates to ensure reproducibility of our findings, and data from all replicates were included in this study and incorporated into our analysis. NAPA was performed using cells from a SE+ healthy donor with two

	different forms of citrullinated fibrinogen functioning as biological replicates. T cell stimulation, tetramer, and MSD cytokine secretion assays were performed on each patient once due to the limited availability of patient samples, but experiments were performed in multiple independent batches over several days to account for batch effects. In all cases, the data was reproducible between replicates and/or batches, and all performed replicates are included in the manuscript.
Randomization	Donors used in T cell stimulation, tetramer, and MSD cytokine secretion assays were separated into experimental groups based on disease status (RA vs HC), antibody status (APCA positive vs negative for RA patients), and HLA allele status (SE allele positive). These groups were selected to test citrullination-dependent T cell epitopes in patients with anti-citrulline immune responses compared to disease controls without anti-citrulline immune responses and healthy controls. All donors were selected for the presence of at least one RA-associated SE alleles to ensure comparable peptide-HLA binding capabilities, and at least one donor from each group was run on each day to account for batch effects.<br><br>Randomization was not relevant to the remaining experiments as samples were not separated into experimental groups.
Blinding	For mass spectrometry experiments (proteolytic mapping and NAPA), the samples were provided to the mass spectrometrists in a blinded fashion with only replicates indicated. Peptides were not provided to ProImmune in a blinded fashion for peptide–HLA-DR binding affinity analysis due to the necessity of providing full peptide sequences (native versus citrullinated). For T cell stimulation and tetramer studies, investigators were not blinded to group allocation due to the necessity for patient-specific cell collection on specific days; however, the same flow cytometry gating strategies were applied to all donors regardless of group to ensure unbiased analysis. Investigators were blinded to patient group when performing the MSD cytokine secretion assays.

Reporting for specific materials, systems and methods

We require information from authors about some types of materials, experimental systems and methods used in many studies. Here, indicate whether each material, system or method listed is relevant to your study. If you are not sure if a list item applies to your research, read the appropriate section before selecting a response.

Materials & experimental systems

n/a	Involved in the study
☐	☒ Antibodies
☒	☐ Eukaryotic cell lines
☒	☐ Palaeontology and archaeology
☒	☐ Animals and other organisms
☒	☐ Clinical data
☒	☐ Dual use research of concern

Methods

n/a	Involved in the study
☒	☐ ChIP-seq
☐	☒ Flow cytometry
☒	☐ MRI-based neuroimaging

Antibodies

Antibodies used	The natural antigen processing assay utilized 20 ug per sample of the BioLegend anti-HLA-DR antibody, clone L243 (1:10 dilution, cat # 307602) for HLA-DR immunoprecipitation.<br><br>10 µg/ml GeneTex anti-human CD40 blocking antibody, clone G28.5 (1:1,300 dilution, cat # GTX14148) was used for T cell stimulation assays.<br><br>The following antibodies were used for flow cytometry in the T cell stimulation assays:<br><br>1:20 BD Pharmingen PE mouse anti-human CD154, clone MR1/RUO (cat # 555700)<br>1:100 BioLegend BV510 mouse anti-human CD3, clone UCHT1 (cat # 300448)<br>1:80 BD Pharmingen PacBlue mouse anti-human CD4, clone RPA-T4 (cat # 558116)<br>1:100 BD Pharmingen PerCP-Cy5.5 mouse anti-human CCR6, clone 11A9 (cat # 560467)<br>1:80 BioLegend AF488 mouse anti-human CXCR3, clone G025H7 (cat # 353710)<br>1:100 BD Pharmingen PE-Cy7 mouse anti-human CCR4, clone 1G1 (cat # 557864)<br>1:40 BD Biosciences APC-H7 mouse anti-human CD8, clone SK1 (cat # 641400)<br><br>The following antibodies were additionally used for flow cytometry in the tetramer staining assays:<br>1:80 BioLegend PE/Dazzle mouse anti-human CD127, clone A019D5 (cat # 351336)<br>1:80 BioLegend PE/Cy5 mouse anti-human CD25, clone BC96 (cat # 302608)<br>1:20 BioLegend BV650 mouse anti-human CCR7, clone G043H7 (cat # 353234)<br>1:20 BD Biosciences BV605 mouse anti-human CD45RA, clone HI100 (cat# 562886)<br>1:20 BioLegend BV711 mouse anti-human PD-1, clone EH12.2H7 (cat # 329928)
Validation	All antibodies used in this study were validated by the respective manufacturers (links provided below) and were titrated before use for us for our specific applications and/or utilized in previous publications by our group (Tiniakou et al. 2020, DOI: 10.1002/art.41248; Fava et al. 2016, DOI: 10.1186/s13075-016-0993-2).<br><br>BioLegend anti-HLA-DR antibody, clone L243 (cat # 307602) - https://www.biolegend.com/en-us/products/purified-anti-human-hla-

dr-antibody-792?GroupID=BLG11943

GeneTex anti-human CD40 blocking antibody, clone G28.5 (cat # GTX14148) - https://www.genetex.com/Product/Detail/CD40-antibody-G28-5/GTX14148

BD Pharmingen PE mouse anti-human CD154, clone MR1/RUO (cat # 555700) - https://www.bdbiosciences.com/en-us/products/reagents/flow-cytometry-reagents/research-reagents/single-color-antibodies-ruo/pe-mouse-anti-human-cd154.555700

BioLegend BV510 mouse anti-human CD3, clone UCHT1 (cat # 300448) – https://www.biolegend.com/en-us/products/brilliant-violet-510-anti-human-cd3-antibody-9792

BD Pharmingen PacBlue mouse anti-human CD4, clone RPA-T4 (cat # 558116) – https://www.bdbiosciences.com/en-us/products/reagents/flow-cytometry-reagents/research-reagents/single-color-antibodies-ruo/pacific-blue-mouse-anti-human-cd4.558116

BD Pharmingen PerCP-Cy5.5 mouse anti-human CCR6, clone 11A9 (cat # 560467) – https://www.bdbiosciences.com/en-eu/products/reagents/flow-cytometry-reagents/research-reagents/single-color-antibodies-ruo/percp-cy-5-5-mouse-anti-human-cd196-ccr6.560467

BioLegend AF488 mouse anti-human CXCR3, clone G025H7 (cat # 353710) – https://www.biolegend.com/en-us/search-results/alexa-fluor-488-anti-human-cd183-cxcr3-antibody-7581?GroupID=BLG6097

BD Pharmingen PE-Cy7 mouse anti-human CCR4, clone 1G1 (cat # 557864) – https://www.bdbiosciences.com/en-us/products/reagents/flow-cytometry-reagents/research-reagents/single-color-antibodies-ruo/pe-cy-7-mouse-anti-human-cd194.557864

BD Biosciences APC-H7 mouse anti-human CD8, clone SK1 (cat # 641400) – https://www.bdbiosciences.com/en-us/search-results?searchKey=641400

BioLegend PE/Dazzle mouse anti-human CD127, clone A019D5 (cat # 351336) – https://www.biolegend.com/ja-jp/products/pe-dazzle-594-anti-human-cd127-il-7ralpha-antibody-9790

BioLegend PE/Cy5 mouse anti-human CD25, clone BC96 (cat # 302608) – https://www.biolegend.com/en-gb/products/pe-cyanine5-anti-human-cd25-antibody-617

BioLegend BV650 mouse anti-human CCR7, clone G043H7 (cat # 353234) – https://www.biolegend.com/en-us/products/brilliant-violet-650-anti-human-cd197-ccr7-antibody-8509

BD Biosciences BV605 mouse anti-human CD45RA, clone HI100 (cat# 562886) – https://www.bdbiosciences.com/en-us/products/reagents/flow-cytometry-reagents/research-reagents/single-color-antibodies-ruo/bv605-mouse-anti-human-cd45ra.562886

BioLegend BV711 mouse anti-human PD-1, clone EH12.2H7 (cat # 329928) - https://www.biolegend.com/en-us/search-results/brilliant-violet-711-anti-human-cd279-pd-1-antibody-7945

Flow Cytometry

Plots

Confirm that:

☒ The axis labels state the marker and fluorochrome used (e.g. CD4-FITC).

☒ The axis scales are clearly visible. Include numbers along axes only for bottom left plot of group (a 'group' is an analysis of identical markers).

☒ All plots are contour plots with outliers or pseudocolor plots.

☒ A numerical value for number of cells or percentage (with statistics) is provided.

Methodology

Sample preparation

For T cell stimulation assays, cryopreserved PBMCs from ACPA+SE+ RA patients (n=10), ACPA-SE+ RA patients (n=8), and SE+ healthy controls (n=10) were thawed, and 1-1.5x10^6 cells were plated per well in a 96-well plate in RPMI culture medium supplemented with 5% Human AB serum (Sigma) and allowed to rest for 8 hours at 37ºC in 5% CO2. PBMCs were then pre-incubated with anti-human CD40 blocking antibody (10 µg/ml G28.5, BioLegend) for 15 minutes prior to stimulation with 2.5 µM of each candidate fibrinogen peptide (>95% purity, synthesized by Elim Pharmaceuticals) or with media alone for 18 hours at 37ºC. The cells were then stained for flow cytometry using the cocktail of antibodies described in the manuscript.

For MHC class II tetramer binding assays, cryopreserved PBMCs from ACPA+SE+ RA patients (n=18), ACPA-SE+ RA patients (n=10), and SE+ healthy controls (n=10) were thawed, and 1-1.5x10^6 cells per condition were then stained for flow cytometry with the MHC class II tetramers (PE- or APC-conjugated) in RPMI culture medium supplemented with 2% fetal bovine serum, 50 nM Dasatinib, and 0.1% sodium azide for 2 hours at 37ºC in 5% CO2. The cells were then stained for flow cytometry using the cocktail of antibodies described in the manuscript.

Instrument

BD FACS Aria II (BD Biosciences) or 5L (five-laser) Cytek Aurora (Cytek Biosciences)

Software	FACSDiva version 6.0 (BD Biosciences) or SpectroFlo (Cytek Biosciences) software were used to collect the data, and FCS Express 7 version 7.12.0009 (De Novo Software) was used for data analysis.
Cell population abundance	Cells were not sorted for this work. Our target analysis population of activated T cells was present at a frequency of 0.67 to 1.0 of CD4+ T cells and is in the expected range for activated T cells based on previously published studies by our group and others.
Gating strategy	Lymphocytes were first gated based on FSC and SSC, then doublets were excluded by sequential FSC H vs. W and SSC H vs. W gating. Live cells were then identified by their exclusion of our live/dead stain (1:300 dilution, Molecular Probes). CD3+ T cells were then gated then CD4 vs. CD8 plotting was performed. The CD4+ CD8- T cells were gated and the % CD154-positive or tetramer-positive cells were quantified and reported. For T helper subset analysis, CCR6+ and CCR6- cells were gated, and the following parameters were used: (CCR6-CCR4+CXCR3-), Th2 (CCR6-CCR4-CXCR3+), Th17 (CCR6+CCR4-CXCR3+), and Th1-17 (CCR6+CCR4+CXCR3-). For effector phenotype analysis, the following parameters were used: T regulatory cells (Tregs; CD127loCD25hi), naïve T cells (Tnaive; CD45RA+CCR7+), effector memory T cells (Tem; CD45RA-CCR7-), central memory T cells (Tcm; CD45RA-CCR7+), and effector memory RA T cells (Temra; CD45RA+CCR7-).

☒ Tick this box to confirm that a figure exemplifying the gating strategy is provided in the Supplementary Information.
