# Supplementary material for: Citrullination modulates antigen processing and presentation by revealing cryptic epitopes in rheumatoid arthritis
Source: Nat Commun. 2023 Feb 24;14:1061. doi: 10.1038/s41467-023-36620-y (PMC9958131; doi:10.1038/s41467-023-36620-y)
Supplement: Supplementary file 4 — Description of Additional Supplementary Files [file 41467_2023_36620_MOESM4_ESM.docx]

**Description of Additional Supplementary Files**

Supplementary Data 1

Description: Raw mass spectrometry data for proteolytic mapping of fibrinogen, vimentin, and hnRNP A2/B1 by cathepsins B, S, and H (sheets 1-6); citrullination site mapping of fibrinogen and vimentin by trypsin digestion (sheets 7-8); and the natural antigen processing assay with native, PAD2-citrullinated (PAD2-cit), and PAD4-citrullinated (PAD4-cit) fibrinogen (sheets 9-11).
